# Tooth regeneration in animals. A systematic review

**DOI:** 10.4317/medoral.27269

**Published:** 2025-08-16

**Authors:** José Bartolomé-Lechuga, Lucía Hernando-Calzado, Carlos Manuel Cobo-Vázquez, Javier Sanz-Alonso, Juan López-Quiles, Cristina Madrigal-Martínez-Pereda

**Affiliations:** 1 ORCID: 0009-0008-8384-3068. Department of Dental Clinical Specialties, Faculty of Dentistry, Complutense University of Madrid, Spain; 2 ORCID: 0000-0002-7821-2869. Department of Dental Clinical Specialties, Faculty of Dentistry, Complutense University of Madrid, Spain; 3 ORCID: 0000-0002-9263-5972. Department of Dental Clinical Specialties, Faculty of Dentistry, Complutense University of Madrid, Spain; 4 ORCID: 0009-0001-1555-3535. Department of Dental Clinical Specialties, Faculty of Dentistry, Complutense University of Madrid, Spain; 5 ORCID: 0000-0002-5817-0526. Department of Dental Clinical Specialties and Surgical and Implant Therapies in the Oral Cavity Research Group, Faculty of Dentistry, Complutense University of Madrid, Spain; 6 ORCID: 0000-0001-8341-6762. Department of Dental Clinical Specialties and Surgical and Implant Therapies in the Oral Cavity Research Group, Faculty of Dentistry, Complutense University of Madrid, Spain

## Abstract

**Background:**

Methods for creating bioengineered replacement teeth benefit from a detailed understanding of the molecular signaling networks that regulate the development of natural teeth. In oral and craniofacial research, spheroid cultures have been explored, various studies on organoids, such as those of salivary glands, taste buds, and teeth, are being conducted. The aim of this review is to provide a comprehensive overview of the current knowledge on dental regeneration.

**Material and Methods:**

This review was registered in PROSPERO (CRD 646053) ad performed following PRISMA guidelines. An electronic search was conducted following the PICO question “In animals (P) subjected to bioengineering techniques (I), is successful dental regeneration achieved (O)?” For evaluating risk of bias, the Arrive scale and the JBI adapted for Quasi-experimental studies tools were used.

**Results:**

A total of 83 articles on dental regeneration from the past 5 years were reviewed, and 4 articles that met the selection criteria were included. The studies describe complete dental regeneration in animal models by stimulating genes such as Wnt10a, Bmp6, Grem2a and the identification of genes and antibodies influencing BMP and Wnt signaling pathways (Sox-2), as well as the expression of key factors such as FGF.

**Conclusions:**

The development of signaling pathways in dental formation has advanced, yet many uncertainties persist, particularly in the regeneration of complete teeth. Despite progress with animal models and genetic editing, identifying suiTable cellular sources and understanding the key genes involved remain essential for future clinical applications.

** Key words:**Dentogenesis, amelogenesis, dentinogenesis, cementogenesis, drug release materials, scaffolds, odontogenic cells, stem cells, whole-tooth regeneration.

## Introduction

Dental absences are a common health condition affecting millions of people. Current dental repair treatments include fillings for caries, endodontic treatments for pulp necrosis, and dental implants to replace missing teeth, all of which rely on the use of synthetic materials. In contrast, the fields of tissue engineering and regenerative medicine (TERMD) employ biologically based therapeutic strategies for the regeneration of vital tissues, thus holding the potential to regenerate living tissues. Methods for creating bioengineered replacement teeth benefit from a detailed understanding of the molecular signaling networks that regulate the development of natural teeth (1).

Tooth development, also known as odontogenesis, begins through interactions between two types of dental tissues: the dental epithelium (DE) and the dental mesenchyme (DM), similar to the interactions between epithelial/mesenchymal cells that guide the formation of many other ectodermal organs, such as hair, sweat glands, and nails (2). A deep understanding of the molecular mechanisms that regulate the natural development of teeth and the differentiation of dental stem cells (DSC) is essential for designing effective approaches for the regeneration of adult dental tissues, including whole teeth. Whole organ regeneration is a feature shared by teeth and other epithelial appendages. Whether the regeneration is cyclical and programmed, or induced by injury or wear, almost all types of epithelial appendages undergo complete organ regeneration (3).

Despite significant differences in their basic compositions as mature organs, various epithelial appendages demonstrate numerous genetic and developmental similarities, which in some cases suggest a profound or even direct homology between subsets of these organs. Since most epithelial appendages exhibit complete organ regeneration, it is hypothesized that these regenerative processes are driven by shared genetic networks.

Spheroid culture is a unique method in which adherent cells are suspended to form cell clusters, unlike conventional flat (two-dimensional, 2D) culture in plates (4). In oral and craniofacial research, spheroid cultures have been explored for salivary gland epithelial cells (4), pulp-derived stem cells (5,6), periodontal ligament-derived stem cells (7,8) and spheroids derived from oral mucosa (9,10). Various studies on organoids, such as those of salivary glands, taste buds, and teeth, are being conducted in the oral and craniofacial regions (11). The aim of this review is to provide an overview of the current knowledge on dental regeneration based on the potential of dental stem cells and address unresolved issues for future research.

## Material and Methods

- Research question

To address the goal of this study, a systematic literature review was conducted following the PRISMA (Preferred Reporting Items for Systematic Reviews and Meta-Analyses) guidelines (12). The protocol was registered in the International Prospective Register of Systematic Reviews (PROSPERO) (CRD 646053). The following research question was established: "What bioengineering technique is successfully employed in dental regeneration?" The P.I.C.O.S. (Population, Intervention, Comparison, Outcome, Study Design) question is defined as: In animals (P) subjected to bioengineering techniques (I), is successful dental regeneration achieved (O)?

- Eligibility Criteria

Inclusion Criteria

1. Articles published from 2010, available in full text in English and/or Spanish.

2. Randomized clinical trials (RCT), prospective clinical trials (CCT), observational studies (retrospective and prospective studies), case series, and case reports.

3. Studies that describe complete dental regeneration. Defining complete dental regeneration as the orderly formation of enamel, dentin, and pulp, exhibiting a morphology similar to the original structure.

Exclusion Criteria

1. Studies describing partial dental regeneration or dental structures.

2. Articles studying the generation of permanent teeth after the eruption of deciduous teeth.

3. Articles on regeneration/recovery of vitality in avulsed teeth.

4. Studies related to periodontal regeneration.

Search strategy

Electronic database PubMed (MEDLINE) was searched for articles published in English or Spanish between 1st January 2010 to 1st December 2024. The search strategy for PubMed was a combination of MeSH terms and keywords in advanced mode without filters: ("tooth"[MeSH Terms] AND ("metabolism"[MeSH Terms]) AND "regeneration"[MeSH Terms] OR "replantation"[MeSH Terms]). The search was supplemented by manually reviewing the references cited in the selected articles and in similar review articles.

- Article Selection

Two independent reviewers (J.B.L. and C.C.V.) conducted the systematic selection of articles. The review process involved evaluating the title, followed by the abstract, and finally assessing the full-text articles to determine if they met the inclusion criteria. In case of disagreement, a third reviewer (J.L.Q.) was consulted to determine the inclusion or exclusion of articles. Duplicates were detected using the Zotero tool (Center for History and New Media, George Mason University, Virginia, USA). The agreement on study inclusion between reviewers was assessed using the Kappa index, which was categorized as slight (0.00 to 0.20), fair (0.21 to 0.40), moderate (0.41 to 0.60), good (0.61 to 0.80), or excellent (0.81 to 1.00).

- Data Collection and Synthesis of Results

Information from each study was recorded using Microsoft Excel 2019 (Microsoft Corporation, Redmond, Washington, USA). The primary variable studied was dental regeneration. Secondary variables included study design and measured variable.

- Quality Analysis of Studies

The quality of animal studies was analyzed using Arrive Scale (13). This scale includes ten items: study design, sample size, inclusion and exclusion criteria, randomization, blinding, outcome measures, statistical methods, experimental animals, experimental procedures and results. The Critical Appraisal Checklist from the Joanna Briggs Institute (JBI) was used for *in vitro* studies, consisting of nine questions that evaluate the internal validity as well as the statistical conclusion validity (14).

By applying the inclusion and exclusion criteria, a total of 4 articles were analyzed. The article search and selection process is represented in the PRISMA flow diagram. The Kappa index was calculated (α = 1) with an excellent level of agreement among authors.

## Results

**Figure 1:**
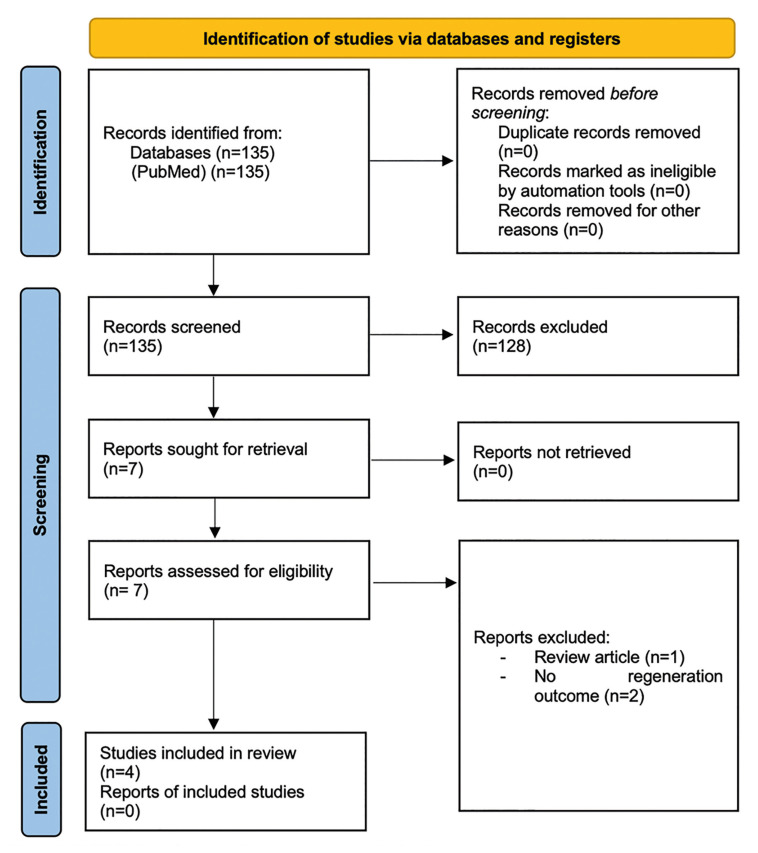
PRISMA flow diagram of the screening and selection process.

The search identified a total of 135 articles in the PubMed database over the last 15 years. By title, 18 articles were included for meeting the inclusion criteria, and after reading the abstract, 7 were accepted for analysis. A total of 4 articles met the selection criteria and were included in this review. The excluded articles were due to the following reasons: not being primary or experimental studies, dentin regeneration in caries, regeneration of permanent teeth after deciduous teeth eruption, regeneration and recovery of vitality in erupted permanent teeth, regeneration of cementum in replanted teeth, and regeneration of periodontal defects. The flow diagram describes the search and selection process (Fig. 1).

Of the 4 articles included in this review, 2 achieved complete dental regeneration, those by Tyler *et al*. (3) and Murashima-Suginami *et al*. (15). Both studies were conducted on animal models, fish and mice/postnatal ferrets, respectively, and both share the common BMP signaling pathway (Table 1, Table 2, Table 3).

Tyler *et al*. (3) experimented with the genes Wnt10a, Dkk2, Bmp6, and Grem2a, hypothesizing that they could be involved in the regulation of tooth organ development or regeneration. They found that three of the four studied ligand-coding genes were necessary for normal primary tooth development in these species of fish: Wnt10a, Bmp6, and Grem2a. They aimed to find a temporally inducible genetic system that could test the effects of Wnt and BMP ligands and inhibitors secreted during the final stages of development without interfering with the initiation of the dental field and early differentiation of primary teeth.

By using heat-shock-induced transgene overexpression in subadult and adult fish, they combined heat-shock treatments of the selected four genes with a two-color pulse-chase bone staining protocol, allowing them to classify each tooth as new or retained. Using 3 to 5 animals per gene, they obtained over 30 dental germs per adult or subadult stickleback fish, supporting their hypothesis (3).

On the other hand, Murashima-Suginami A *et al*. (15) investigated the phenotypic changes in an Msx1 −/− /USAG-1 −/− mouse model with congenital dental agenesis and supernumerary teeth.

To investigate whether the inhibition of USAG-1 function rescues congenital dental agenesis, five monoclonal USAG-1 antibodies were purified from mouse (#12, #16, #37, #48, and #57) using a recombinant human bioactive USAG-1 protein derived from *Escherichia coli* as an antigen and USAG-1 −/− mice. It was suggested that USAG-1 inhibits Wnt and BMP signaling by binding directly to BMP and the Wnt co-receptor LRP5/6. Therefore, these five antibodies were classified into three different groups based on their ability to interfere with binding to both BMP and Wnt (#57), BMP (#12 and #37), or Wnt (#16 and #48). All antibodies were confirmed to bind to both mouse and human recombinant USAG-1 proteins, although #16 and #48 showed low affinity. These results allowed further investigation of the function of USAG-1 concerning BMP and Wnt signaling pathways for determining the number of teeth (15).

To confirm that the neutralizing activity of USAG-1 affects BMP signaling to generate a complete tooth in a non-rodent model, antibody #37 was systemically administered to postnatal ferrets with deciduous and permanent teeth. The formation of supernumerary teeth in maxillary incisors similar to third dentition was observed, though it required five times the concentration, three administrations of antibody #37, and immunosuppression. The supernumerary tooth likely resembled the permanent incisor and was located on the lingual side of the permanent teeth, although it appeared to be growing a shorter root. Therefore, this supernumerary incisor could be classified as third dentition (15).

Fan Shao *et al*. (16) identified key transcription factors for both embryonic and postnatal transcription, proposing multiple markers for dental epithelial stem cells (DESC), including Sox2, Lgr5, Gli1, Lrig1, Bmi1, and Ptch1. Among these, Sox2 is the best-characterized marker, as it has been shown to give rise to all epithelial lineages in the tooth. Using an Sox2-GFP mouse line, they identified genes that are enriched or positively regulated in Sox2-DESC (16).

Wenwen Guo *et al*. (17) examined the dynamic expression of representative ligands and receptors of FGF, as well as antagonists, in the lower third molar at E40, E50, and E60. They demonstrated that FGF3 plays an important role in tooth development planning and morphogenesis, revealing similarities and differences between mice, pigs, and humans. In humans, FGF3 is predominantly expressed in the mesenchyme, particularly in the preodontoblastic and odontoblastic layers at the late stage, and these findings were similar. The results indicated that FGF plays a critical role in cusp patterning and odontoblast differentiation. These receptors, associated with gene expression and functional networks in miniature pigs, require deeper analysis. It is believed that FGF4 and FGF9 expressed in epithelial cells maintain FGF3 expression in the dental mesenchyme, further regulating the proliferation and morphogenesis of epithelial cells. Specifically, FGF3 and FGF7 stimulate the proliferation of inner and outer enamel epithelial cells, respectively (17). The spatiotemporal distribution of FGF gradients and sprouty molecules during odontogenesis is determined, and epithelial-mesenchymal interactions could be modulated by adjusting the FGF pathway to promote cusp patterning and dental mineralization (17).

After evaluating the methodological quality of animal studies using the Arrive Scale (13), it was determined that the studies conducted by Fan Shao *et al*., Murashima *et al*. and Tyler *et al*., had a medium risk of bias (Table 2). The quality analysis of Wenwen Guo *et al*. was performed according to the Joanna Briggs Institute (JBI) Critical Appraisal Checklist for quasi-experimental studies (14) (Table 3).

## Discussion

The regeneration of a complete tooth as an organ replacement therapy is considered the ultimate goal in regenerative dentistry. For patients, this therapeutic option could represent a dream for replacing decayed or lost teeth, overcoming the need for prosthodontic or implantological treatments that use artificial substitutes. The generation of complete teeth could be achieved as a hybrid strategy, for example, by combining biologically created tissue compartments, such as the periodontal ligament or a dental crown, with a metallic or ceramic implant, or by combining a biologically regenerated dental root ("bio-root") with a prosthetic crown. In the coming years, efforts to create a complete tooth from only cells and tissues ("bio-tooth") are likely to be the focus. However, despite all the efforts and results achieved in basic and translational research, this approach remains a challenge (18).

In the context of tooth evolution, a genetic approach to generate complete teeth could be an option in the distant future. In mammals, many species, including humans, are only diphyodonts with the ability to form a second dentition, or even monophyodonts like the mouse (19,20,21). The revitalization of odontogenic potential for the regeneration of lost teeth may be an interesting approach to induce the formation of teeth *in vivo* in adults. A prerequisite for dental replacement is the existence of a successional dental lamina (SDL) that has the ability to induce odontogenesis. Even in monophyodont animals, a rudimentary SDL has been identified. Furthermore, in humans, rudimentary laminae are preserved, which could be responsible for a third dentition, although this has been observed very rarely. At the molecular level, tooth replacement is regulated by signaling pathways. The stem cells in the SDL express Sox2, which is initiated by the Wnt/β-catenin pathway and interacts with BMP signaling (21).

It has been discussed that the deregulation of Wnt signaling is important for the deactivation of the rudimentary SDL, as seen in mice. Therefore, revitalization through the stabilization of Wnt signaling using appropriate factors or genes could be a strategy for inducing tooth growth in the future (21,22).

In humans, there are many obstacles, such as the fact that dental germs may not be easily accessible, but ethical and legal restrictions should also be considered. An alternative could be the use of adult stem cells (23,24).

The method for creating complete teeth would be the use of autologous dental cells from patients seeking dental regeneration. Different strategies for bioengineering complete teeth have been developed with these cells. One idea was to combine adult stem cells with progenitor cells from the embryonic tooth. Adult stem cells should have odontogenic competence and should function as a "dental inducer" when combined with mesenchymal cells or express dental mesenchymal competence when combined with dental epithelium (18). Smith *et al*. proposed that the dental lamina provides a protected environment for odontogenic stem cells, which serve as a reservoir of tooth-forming potential, and their position determines the site of future tooth development (25).

Dental epithelial stem cells were first defined in the context of the continuous growth of mouse incisors (26), although they were discovered much earlier as slow-cycling cells by Smith and Warshawsky (27). During tooth development, stem cells reside in the niche called the cervical loop, where they actively participate in crown formation until the start of root formation, which coincides with the loss of stem cells and the deactivation of the niche (28). Therefore, in teeth that develop roots, like mouse molars and all human teeth, epithelial stem cells with regenerative potential are lost before tooth development is completed (29). In continuously growing teeth, such as mouse incisors, stem cells and the niche remain active throughout the animal's life (29). Expression analysis of Sox2, one of the markers of dental epithelial stem cells, has indicated that most cells within the dental lamina of mice express this marker, while in the placode, Sox2+ cells are restricted to the lingual side opposite the initiation knot, which governs their proliferation in the bud (30,31).

However, it is still not known whether Sox2+ cells from the dental lamina migrated to the lingual side of the placode or if the expression of Sox2 in this location was recently acquired. While it is not known whether Sox2+ cells in the placode represent stem cells, studies in mice and sharks have implicated that successor teeth are formed from Sox2+ stem cells of the predecessor tooth (31,32).

A small number of so-called key transcription factors is sufficient to establish the genetic expression programs that define cellular identity. Additionally, it has been demonstrated that overexpressing a few key transcription factors is enough to direct cell lineage.

Fibroblast growth factor (FGF) family proteins mediate inductive interactions between dental epithelium and dental mesenchyme during successive stages of tooth formation. Growth factor signaling is crucial during the development of dental epithelium and mesenchyme. Wenwen Guo *et al*. have investigated tooth development and the cascade initiation of permanent molars in the third deciduous molars of miniature pigs, characterizing dynamic gene expression profiles associated with the fibroblast growth factor signaling pathway (17).

In the study of three-spined stickleback fish and zebrafish, it was determined that stimulation of the BMP pathway or inhibition of the Wnt pathway would increase the rate of tooth replacement, while stimulation of the BMP pathway or inhibition of the Wnt pathway would inhibit regeneration (3). From the four genes studied (Wnt10a, Bmp6, Grem2a and Dkk2), they found that Wnt10a, Bmp6 and Grem2a are necessary for the normal development of teeth. These four studies genes also show expression involvement between epithelial and mesenchymal germ layers, resembling the expression domains previously reported for mammalian orthologs. Their results highlight a previously unknown flexibility in the regulation of tooth replacement: it is possible to significantly accelerate or slow down the tooth replacement cycle in both species of fish studied here. These results define important regulatory mechanisms that affect tooth development and regeneration. (3)

Similar to Tyler *et al*. (3), Murashima-Suginami *et al*. (15) analyze the BMP signaling pathway via Anti-USAG-1. The deficiency of the uterine sensitization-associated gene 1 (USAG-1) leads to increased bone morphogenetic protein (BMP) signaling. Interactions involving positive and negative loops between BMP, fibroblast growth factors, Sonic hedgehog, and Wnt pathways regulate the morphogenesis of individual teeth. USAG-1 is a bifunctional protein that antagonizes BMP and Wnt, the two essential signaling molecules for tooth development.

The systemic administration of neutralizing antibodies against USAG-1, which mainly interfere with BMP signaling (#37 and #57), rescued dental agenesis in EDA1-deficient mice and led to the efficient formation of a complete tooth in a dose-dependent manner in wild-type mice. Previously, the identification of antibodies that could promote dental regeneration had not been reported. The generated antibodies neutralized USAG-1's antagonistic action on BMP signaling. However, the participation of Wnt signaling cannot be excluded based on these findings, as several mice were stillborn or did not survive. Therefore, further experiments, such as epitope classification involving a greater number of USAG-1 neutralizing antibodies and detailed analyses of recombinant USAG-1 protein epitopes, are necessary (15).

The present study has several limitations that should be considered when interpreting the results. Firstly, the number of published articles on the subject is limited, reducing the availability of evidence. Moreover, among the few available studies, the majority are reviews rather than experimental investigations, which constrains the strength of the findings. Regarding methodological quality, it was observed that several of the included articles do not provide detailed information on the sample used, participant follow-up, or complications encountered during the process, making it difficult to assess the external validity of the results. These methodological shortcomings, along with inadequate follow-up, restrict the ability to draw firm and generalizable conclusions on the subject.

## Conclusions

The development of signaling pathways in tooth formation has advanced, but many questions remain unsolved. Although mouse models and genetic editing have been useful, limitations in cellular analysis techniques hinder progress. To regenerate complete teeth, it is crucial to identify suiTable cell sources and understand the genes responsible for tooth formation. While the use of dental stem cells in clinical applications remains a challenge, a promising future for the regeneration of complete teeth and dental tissues is anticipated.

## Figures and Tables

**Table 1:** Description of selected studies.

Author	Year of Publication	Study Design	Measured Variable	Dental Regeneration
Fan Shao et al.	2023	Animal study - Mice	Sox2 as a marker for Dental Epithelial Stem Cells (DESC)	No
Tyler A. et al.	2023	Animal study - Fish (Three-spined stickleback and zebrafish)	WNT and BMP signaling pathways	Yes
Wenwen Guo et al.	2022	Animal study - Miniature pigs	FGF signaling in Morphogenesis and Odontogenesis	No
Murashima-Suginami A et al.	2021	Animal study - Mice/Hurons (Postnatal)	Anti-Usag1 in BMP signaling pathway	Yes

**Table 2:** Quality of animal studies analyzed using Arrive Scale.

ITEM		QUESTION	ANSWER		
			Fan Shao et al. (2023)	Murashima et al. (2021)	Tyler et al. (2023)
1	Study design	Are all experimental and control groups clearly identified?	No	No	No
		Is the experimental unit (e.g. an animal, litter or cage of animals) clearly identified?	Yes, for at least one experiment	Yes, for at least one experiment	Yes, for at least one experiment
2	Sample size	Is the exact number of experimental units in each group at the start of the study provided (e.g. in the format ‘n=')?	No	Yes, for at least one experiment	Yes, for at least one experiment
		Is the method by which the sample size was chosen explained?	No	Yes, for at least one experiment	Yes, for at least one experiment
3	Inclusion and exclusion criteria	Are the criteria used for including and excluding animals, experimental units, or data points provided?	No	No	No
		Are any exclusions of animals, experimental units, or data points reported, or is there a statement indicating that there were no exclusions?	No	No	No
4	Randomisation	Is the method by which experimental units were allocated to control and treatment groups described?	No	No	No
5	Blinding	Is it clear whether researchers were aware of, or blinded to, the group allocation at any stage of the experiment or data analysis?	No	No	No
6	Outcome measures	For all experimental outcomes presented, are details provided of exactly what parameter was measured?	Yes, for at least one experiment	Yes, for at least one experiment	No
7	Statistical methods	Is the statistical approach used to analyse each outcome detailed?	No	Yes, for at least one experiment	Yes, for at least one experiment
		Is there a description of any methods used to assess whether data met statistical assumptions?	No	Yes, for at least one experiment	Not applicable
8	Experimental animals	Are all species of animal used specified?	Yes, for at least one experiment	Yes, for at least one experiment	Yes, for at least one experiment
		Is the sex of the animals specified?	Yes, for at least one experiment	Yes, for at least one experiment	No
		Is at least one of age, weight or developmental stage of the animals specified?	Yes, for at least one experiment	Yes, for at least one experiment	No
9	Experimental procedures	Are both the timing and frequency with which procedures took place specified?	No	Yes, for at least one experiment	Yes, for at least one experiment
		Are details of acclimatisation periods to experimental locations provided?	No	Yes, for at least one experiment	Yes, for at least one experiment
10	Results	Are descriptive statistics for each experimental group provided, with a measure of variability (e.g. mean and SD, or median and range)?	Not applicable	Not applicable	Not applicable
		Is the effect size and confidence interval provided?	Not applicable	Not applicable	Yes, for at least one experiment

**Table 3:** Quality of in vitro studies using Critical Appraisal Checklist from the Joanna Briggs Institute (JBI).

				Wenwen Guo et al. (2022)			
				Yes	No	Unclear	N/A
INTERNAL VALIDITY	Bias related to temporal precedence	1	Is it clear in the study what is the "cause" and what is the "effect" (i.e. there is no confusion about which variable comes first)?	-	-	-	X
	Bias related to selection and allocation	2	Was there a control group?	-	X	-	-
	Bias related to confounding factors	3	Were participants included in any comparisons similar?	X	-	-	-
	Bias related to administration of intervention/exposure	4	Were the participants included in any comparisons receiving similar treatment/care, other than the exposure or intervention of interest?	-	-	-	X
	Bias related to assessment, detection and measurement of the outcome	5	Were there multiple measurements of the outcome, both pre and post the intervention/exposure?	-	-	-	X
		6	Were the outcomes of participants included in any comparisons measured in the same way?	-	-	-	X
		7	Were outcomes measured in a reliable way?	X	-	-
	Bias related to participant retention	8	Was follow-up complete and if not, were differences between groups in terms of their follow-up adequately described and analyzed?	-	-	X
STATISTICAL CONCLUSION VALIDITY		9	Was appropriate statistical analysis used?	X	-	-	-
